# Psychosocial oncology in Sub-Saharan Africa: Lessons from Ghana

**DOI:** 10.1002/pon.5965

**Published:** 2022-05-24

**Authors:** Hermioni L. Amonoo, Salisu A. Abdul-Rahim, Deborah Atobrah, Dorothy Addo-Mensah, Regina M. Longley, Michelle C. Jacobo, William F. Pirl

**Affiliations:** 1 Department of Psychosocial Oncology and Palliative Care, Dana-Farber Cancer Institute, Boston, Massachusetts, USA; 2 Department of Psychiatry, Brigham and Women’s Hospital, Boston, Massachusetts, USA; 3 Harvard Medical School, Boston, Massachusetts, USA; 4 National Radiotherapy, Oncology, and Nuclear Medicine Center, Korle-Bu Teaching Hospital, Accra, Ghana; 5 Institute of African Studies, University of Ghana, Accra, Ghana; 6 Department of Adult Health, School of Nursing and Midwifery, University of Cape-Coast, Cape-Coast, Ghana

**Keywords:** cancer, case Series, Ghana, low-income country, mental health, palliative care, psycho-oncology, psychosocial wellbeing, sub-Saharan Africa, supportive oncology

## 1 | BACKGROUND: PSYCHOSOCIAL ONCOLOGICAL CARE IN SUB-SAHARAN AFRICA (SSA)

The public health burden of cancer in sub-Saharan Africa (SSA) is escalating with an estimated 2.12 million new cancer cases per year by 2040.^1^ The mortality rate from cancer in SSA is 1.5-4-fold higher than in high income countries (HIC).^2^ Compared to HIC, resources for cancer prevention (e.g., vaccines) and early detection measures (e.g., colonoscopy, mammography, Human Papilloma Virus screening) are also abysmal, resulting in delayed diagnosis and more aggressive, late-stage disease in at least 80% of patients diagnosed with cancer.^3^ Further, robust and timely cancer diagnostics and treatment options are also lacking in SSA.^4,5^ In addition to these systemic challenges and scarcity of oncology clinicians in SSA, poverty, low health literacy, and cultural and psychological factors (e.g., fear regarding the etiology and management of cancer) pose significant barriers to timely oncological care.^6,7^ While much work is desperately needed to enhance early detection, diagnostics, and therapeutics for cancer care in SSA, the unmet psychological needs of patients with cancer are also worthy of consideration and should be tackled.^8^

Robust evidence supports that psychological well-being is essential to navigating the cancer care and treatment cycle.^9,10^ Despite progress to integrate palliative care into oncological care in low-and-middle income countries (LMICs),^11,12^ the focus of palliative care integration has been on pain relief with less emphasis on the psychological aspects of care.^13,14^ Although increased burden of physical symptoms and pain from often more aggressive disease in the setting of limited treatment options contributes significantly to this emotional suffering, data on best ways to assess and manage the psychological needs of the cancer population in SSA is non-existent.^15,16^ Consequently, psychosocial oncology, the cancer specialty that addresses psychological, behavioral, and social concerns of patients with cancer and their caregivers has not been a focus of oncology work in most LMICs.^8^

In the past decade, multidisciplinary groups of clinicians in SSA including psychologists, nurses, and oncologists have used interest groups and societies to coordinate efforts of educating patients and clinicians about the importance of psychosocial oncology in oncological care. One of the first psycho-oncology special interest groups was formed under the African Organization for Research and Training in Cancer (AORTIC) which is the largest professional organization for oncology clinicians and researchers in SSA.^17^ Country-specific organizations registered with the International Psychosocial Oncology Society (IPOS) Federation are in Ghana, Nigeria, and Kenya.^18,19^ Although these psychosocial oncology groups have similar mission statements centered around education, research, and clinical care improvement, there are limited data on the scope of their efforts and how effective they are in accomplishing their goals and mission. In 2019, a group of 20 Ghanaian oncology clinicians (e.g., psychologists, nurses) formed the Psycho-Oncology Society of Ghana (PoSoGH), an independent organization and vehicle to increase awareness and educate the public and clinicians about the psychological aspects of cancer care, which has not been a focus of oncological care in Ghana. Similar to other psychosocial oncology organizations in SSA, PoSoGH is the first organization of its kind in Ghana. The leadership of PoSoGH hoped to use conferences, workshops, and research to improve psychosocial oncology care. In the setting of limited psychosocial oncology training and practitioners in the region, the leadership of PoSoGH aimed to foster global collaborations especially with more established academic institutions. As clinicians and researchers outside SSA raised questions about the nature of psychosocial oncological care in Ghana and SSA, this project was birthed to illustrate the current state of psychosocial oncological care in Ghana and SSA with a discussion of potential strategies and recommendations for bolstering psychosocial oncology services in Ghana and other SSA countries. To our knowledge, this would be the first published project organized by a psychosocial oncology society in SSA in collaboration with clinicians and researchers outside the region.

## 2 | METHODS

To provide an overview of the nature of psychosocial oncology services in SSA, we conducted a scoping review of the literature describing psychosocial oncology services in SSA countries including Ghana. We completed a comprehensive search in MEDLINE/PubMed, Google Scholar, and Google search from June-August 2021 to identify any publication (i.e., not just limited to peer-reviewed articles) on psychosocial oncology in SSA. Keywords related to psychosocial oncology such as ‘psychosocial oncology’, ‘mental health’, or ‘psycho-oncology’ were combined with keywords related to the region such as ‘sub-Saharan Africa’, ‘Africa’, ‘Ghana’, ‘Kenya’, or ‘Nigeria’ or ‘West Africa’. We reviewed all results including peer-reviewed journal articles, dissertations, conference proceedings, and websites. In addition to the scoping review, we also assembled a multidisciplinary group of psychosocial oncology clinicians (H.A., W.P., S.A., M.J.), researchers (H.A., W.P., D.A.), a nurse (D.A.M.), and an anthropologist (D.A.) from the United States and Ghana to first describe the current state of psychosocial oncology in Ghana. Our team not only examined the current structure and resources, but also their own lived experiences of assessing and addressing the psychosocial needs of patients with cancer. In doing so, we strive to illustrate care at the patient level and to provide some context for those who have no experience caring for patients in SSA. Hence, clinicians and psychologists in the group provided representative examples (i.e., via four cases) of patient-level care based on their lived experiences at the National Radiotherapy, Oncology, and Nuclear Medicine Center (NRONMC) at the Korle-Bu Teaching Hospital in Accra, Ghana. Finally, the multidisciplinary group then collaboratively attempted to provide some recommendations to bolster psychosocial oncology clinical care and research in the region, with the caveat that each African country has a unique set of resources and infrastructure for healthcare that may not translate from one context to the next.

## 3 | RESULTS

**FIGURE 1:**
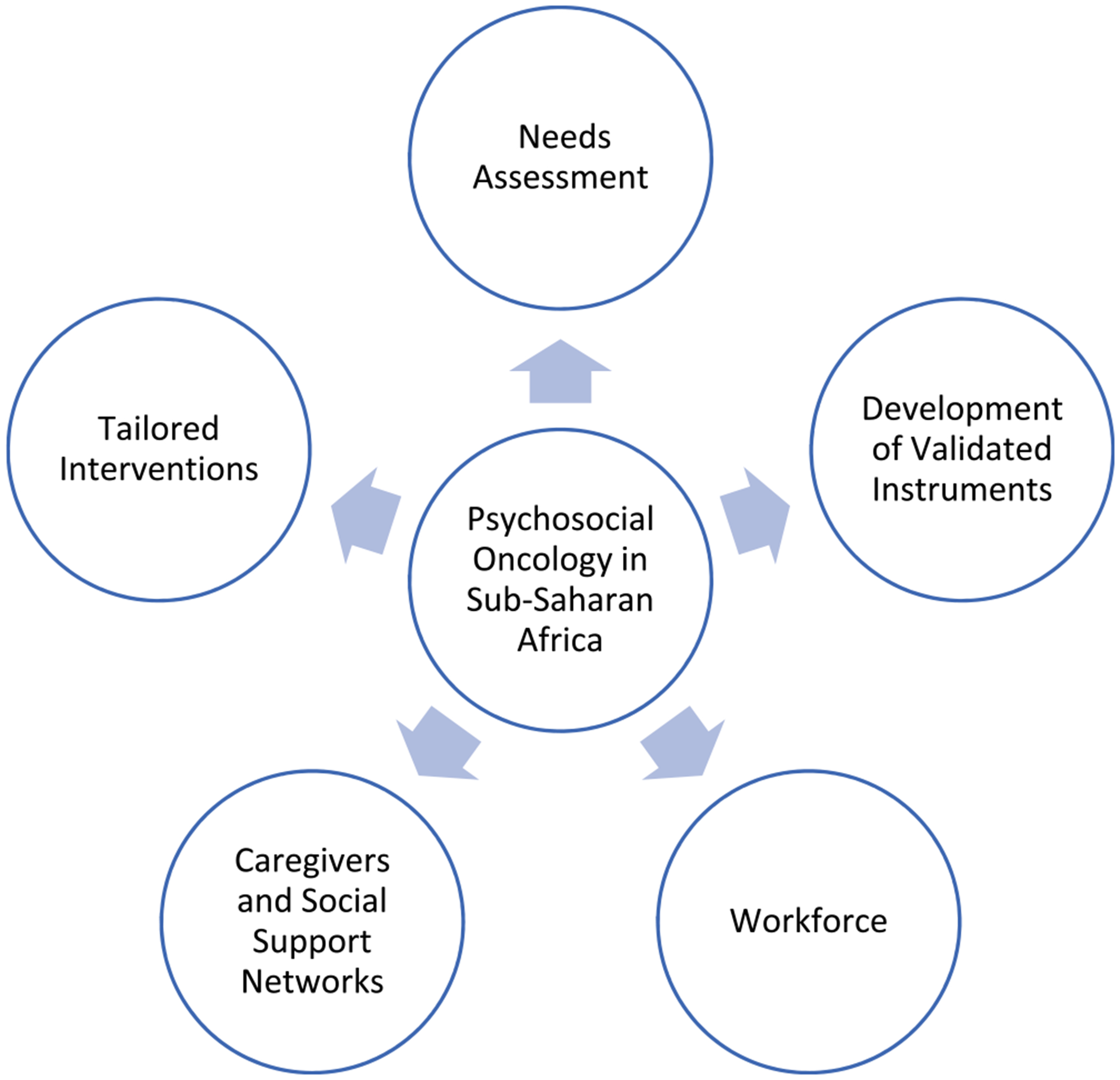
Areas for consideration for psychosocial oncology research in sub-Saharan Africa

Informed by our literature search and expert report, we describe the role of psychosocial oncology societies in the region. We also provide an overview of psychosocial oncological care in SSA informed by anecdotal experiences of experts in Ghana and describe the dearth of psychosocial oncological resources (Figure 1) for the clinical care of patients in SSA.

### 3.1 | Scoping review

We found only four peer-reviewed articles that focused on psychosocial oncology in SSA, see Table 1.^18–21^ While one study described the nature of psychosocial services delivered in Nigeria and the challenges of incorporating psychosocial care in routine oncological care,^19^ another outlined the potential benefits and limitations of social work in the delivery of psychosocial care in SSA.^20^ Although not specifically focused on SSA, another article summarized a plenary at an IPOS annual meeting that addressed the challenges and opportunities to developing psychosocial oncology care in LMIC.^21^ Additionally, one survey study of leaders from the International Federation of Psycho-oncology Societies from 28 countries by Grassi and colleagues showed that psycho-oncology services are underdeveloped in SSA and South East Asia.^22^ Limited literature on the delivery of psychosocial oncology services in SSA underscores the need for more work to incorporate psychosocial oncology in routine cancer care in SSA.

### 3.2 | Psychosocial oncological care in Ghana

SUB-SAHARAN AFRICA constitutes a large geographic region with 46 countries and over 1000 languages characterized by diverse cultures, healthcare systems, and healthcare resources for oncological and psychosocial oncological care.^23^ While we provide clinical anecdotes and expert reports from one country in the region, Ghana, where the unmet psychosocial oncology needs may be applicable to the entire region, we acknowledge that each country has unique characteristics, strengths, and challenges pertaining to healthcare. Ghana, a SSA country in West Africa has a population of approximately 30 million. Of the 22,823 reported cancer cases in 2018, 66% died from their malignancy and cancer is attributed to 17.9% of premature deaths from non-communicable diseases.^24^ Solid malignancies make up a majority of reported cancer cases in Ghana and other SSA countries with cervical and breast cancer being the most prevalent.^25^ As in most LMICs where data on scope of practice and specialty is not robustly obtained and archived, it is difficult to ascertain accurate information on the number and nature of individuals making up the oncology workforce in Ghana and other SSA countries.^26^ While some Ghanaian patients with cancer are cared for by the 12 oncologists in the country, most patients with cancer receive oncological care from non-oncology clinicians including general practitioners, surgeons, or traditional and spiritual healers.^27^ With a physician to patient ratio of 1:10,450 in Ghana, the interaction between physicians and patients with cancer is limited overall.^28^ Hence, the cancer care system is unable to adequately address the psychosocial needs of this population in Ghana and many SSA countries.^28^

Oncology clinicians in Ghana who seek comprehensive cancer treatment for their patients can refer them to the NRONMC at the Korle-Bu Teaching Hospital in Accra, Ghana. About 1500 Ghanaian patients with cancer are referred for cancer care at NRONMC annually. Approximately 27% (*N* = 400) of oncology cases at NRONMC are referred to see one of the two mental health clinicians with master’s level training for psychological evaluation. Half (*N* = 200) of the patients referred for psychological evaluation require psychosocial care and receive an average of five outpatient sessions with a mental health clinician following the initial psychosocial assessment. Based on volume, one mental health clinician is designated to the breast surgical oncology team while the other manages referrals for all patients with non-breast malignancies.

### 3.3 | Lived experience of providing psychosocial oncology care in Ghana

Examples of typical patient cases referred to mental health clinicians at NRONMC, are summarized in Table 2. These cases are not intended to provide clinical guidance for the delivery of psychosocial oncological care in Ghana or SSA but to provide psychosocial oncology clinicians who are not familiar with the region a description of the lived experience of providing psychosocial oncology care in SSA. Notably, the case summaries lack comprehensive patient information typically obtained by psychosocial oncology clinicians in more developed countries with clear standards for clinical encounter documentations and sophisticated medical record systems.^12^ Briefly, most mental health referrals are for evaluation of symptoms of anxiety and depression. Although most patients referred to see a mental health clinician have initiated some cancer treatment (e.g., chemotherapy), they have no prior psychiatric diagnoses or interactions with a mental health clinician. All mental health evaluations are conducted in an outpatient setting. A medical work-up is typically not part of routine psychosocial oncology evaluations. The primary psychosocial treatment offered to all patients is a cognitive behavior therapy (CBT)-based treatment with relaxation exercises depending on patients’ interest. Follow-up psychological care are routinely offered to patients although proximity to the cancer center is a barrier to treatment. Psychopharmacological treatment options are limited – another referral must be made to a local psychiatrist since there are no psychiatrists at cancer centers such as the NRONMC. With pervasive stigma of mental illness in Ghana and SSA,^29,30^ very limited numbers of psychiatrists (e.g., 16 in Ghana and 2 in Liberia), and none with sub-specialty training in psychosocial oncology, most patients with cancer decline a referral to a psychiatrist.

### 3.4 | Recommendations for enhancing psychosocial oncological care in SSA

A nuanced understanding of the psychosocial needs of patients with cancer is essential to exploring viable strategies to address the unmet psychosocial oncology needs in SSA. We provide considerations for enhancing psychosocial oncology care in SSA where formal infrastructures for psychosocial oncology are currently lacking.

### 3.5 | Who should administer psychosocial oncology services?

The psychosocial oncology workforce in SSA is primarily comprised of master’s level psychologists. While nurses and especially psychiatric nurse practitioners can supplement the work done by psychologists, psychiatric nurse practitioners with psychosocial oncology training are non-existent in Ghana and most of SSA. In some settings, oncology nurses have effectively delivered palliative care including some psychological assessments for cancer patients.^11,19^ Although social workers constitute the majority of psychosocial oncology clinicians in HICs, social workers in Ghana and most SSA countries lack training in psychosocial oncology and are not part of routine oncological clinical teams.^20^ With ongoing shortages of oncologists and other specialized oncology clinicians in SSA,^1^ oncology clinicians are not poised to screen and assess for the psychological needs of their patients. Hence, a thorough understanding of the clinicians that care for patients with cancer in general is essential to establishing the workforce needs and opportunities for delivering psychosocial care for the cancer population. An exploration of task-sharing^31^ among a diverse group of clinicians (e.g., nurses, social workers, psychologists, oncologists, general practitioners) for pharmacological and non-pharmacological management maybe especially relevant for the SSA context.

### 3.6 | What types of assessment tools exist and could be tailored to screen and monitor psychological well-being among SSA patients with cancer?

Evidence for best practices for universal distress or psychological well-being screening is growing in HICs.^32^ However, work to identify what is viable for low resource settings is needed – what has worked in HICs may not necessarily translate to SSA or low-resource settings.^33–36^ Most psychological assessments for patients with cancer in SSA incorporate validated instruments, with the Brief Symptom Inventory^37^ being common. While there are several validated instruments to assess both the negative and positive aspects of psychological well-being,^38–40^ very few are part of the routine psychological assessments.^33–36^ With high rates of mobile phone penetration in SSA,^41^ mobile-health technology is already being used to deliver palliative care services and other non-communicable disease management in the African region.^42,43^ Hence, the integration of psychological self-assessment tools with mobile health technology could be another viable way to facilitate the integration of validated psychosocial assessments in the care of the cancer population.^44^

### 3.7 | What are the existing treatment options for psychosocial oncology care in SSA and how might they be best utilized?

Cognitive behavioral therapy frameworks are comprised of: 1) education regarding how maladaptive thoughts, emotions, and behaviors influence distress, 2) approaches to identifying and changing distorted thinking, and 3) coping skills enhancement to manage the stressors along the cancer care continuum, are routinely offered by psychosocial oncology clinicians in SSA.^45,46^ Stress management with mindfulness and relaxation techniques are also frequently offered to patients with cancer in SSA.^47^

Although peer-based interventions have been successfully used and associated with improved patient-reported outcomes (e.g., social support), very few formal peer-based resources exist in SSA.^48,49^ Hence, trained cancer survivors and their caregivers can serve as effective peer mentors who can help address some of the psychological burden of the cancer population.

Integrated palliative care interventions have been effective in improving psychological well-being, reducing symptom burden, and mortality for patients with cancer.^50,51^ Since palliative care interventions facilitate end-of-life care preferences of patients regardless of goals of care or anticipated outcomes, they may be especially important in SSA where most patients present with end-stage disease and treatment options are quite limited. Palliative care interventions have been successfully used in some African countries,^52,53^ and it would be useful to understand barriers to palliative care integration in SSA to determine the role of palliative care in addressing the unmet psychosocial needs of the cancer population.

Since spirituality has been associated with improved adjustment and functioning in a variety of diverse cancer populations,^54,55^ spiritual care may be an existing resource for patients in SSA where the majority of the population are religious (i.e., Christianity or Islam) and devout.^56^ Despite the mixed and limited evidence on the impact of religion and spirituality on psychological well-being of patients, spiritual care may be beneficial in managing psychological distress in SSA.

### 3.8 | How can HICs serve as partners in improving psychosocial oncology care in SSA?

One viable mechanism to bolster psychosocial oncology resources in SSA is via educational partnerships between HICs’ academic institutions with robust psychosocial oncology resources and LMICs. Data on effective ways to foster these partnerships specifically for oncological care is limited. However, the Toronto Addis Ababa Partnership (TAAP), a collaboration between the University of Toronto and the University of Addis Ababa that was instrumental in increasing the number of psychiatrists in Ethiopia over several years, could be explored as a viable model for psychosocial oncology.^57^ TAAP entailed University of Toronto faculty traveling to Addis Ababa to train psychiatrists over several years. Over time, there were enough local psychiatrists to lead their own educational programs. Increased use of virtual platforms such as Zoom Video Communications Inc., San Jose, CA application (zoom.us)^58^ for education purposes during the coronavirus-2019 pandemic further expands the possibilities of such training collaborations and could be a promising model for psychosocial oncology in SSA. While TAAP could only leverage the few faculty with the flexibility to travel to Ethiopia, with virtual platforms, more faculty from different geographic regions could participate in such training efforts.

Additionally, psycho-oncology societies could provide another vehicle for collaboration between SSA and HIC clinicians and researchers. Although existing societies have been effective in bringing together local or national clinicians, most lack the resources and training on cutting edge practices and care delivery in the field. Hence, educational partnerships between these organizations and HIC academic institutions for training of local experts could facilitate their effectiveness in educating patients and other local clinicians about psychosocial oncological care and delivery.

### 3.9 | Clinical implications and suggestions for future research

A lot of work needs to be done to make psychosocial oncology services more robust in Ghana and other SSA countries. We propose four preliminary areas for consideration in addressing psychosocial oncological care in SSA. First, research that describes the nature of psychological needs in the cancer population is necessary to developing population specific supportive oncology interventions. Second, research on the best assessment tools for psychological distress is also essential since most of the current validated instruments were developed in HICs and may not translate well to SSA or LMICs. Third, data on the capacity and limitations of existing psychosocial oncology workforce is scarce. Hence, an understanding of existing workforce infrastructure will be helpful in determining how to effectively train clinicians to address the psychological needs of the cancer population. Fourth, research that examines the most effective pharmacological and non-pharmacological interventions to address the psychological needs of the cancer population in Ghana and other SSA countries will further help to tailor interventions to meet unmet needs.

### 3.10 | Limitations

There are several limitations of this project that warrant discussion. First, we primarily reported on information from peer-reviewed journal publications or publicly available publications from the Internet. Hence, it is possible that we may have missed essential information on the state of psychosocial oncology care in various parts of SSA that have not been published or publicly reported. Second, we only reviewed information that was published in English. Since several SSA countries primarily use languages other than English, we could have also missed information from these countries and settings. Third, our reported clinical anecdotes were from the Ghanaian context, which may not generalize to other SSA countries based on the diversity of healthcare resources and cultural implications across the region.

## 4 | CONCLUSION

In sum, there are several unmet psychosocial oncology needs in the cancer population in Ghana and other SSA countries. Despite the dearth of resources to address the psychological needs of the cancer population, opportunities exist to define what the issues are to inform the development of practical interventions that will improve overall patient outcomes in these settings.

## Figures and Tables

**TABLE 1:** Peer-reviewed articles focused on psychosocial oncology in sub-Saharan Africa

Author, year	Ref.	Population	Design	Significant results
Grassi, 2012	18	Representatives of 29 member countries of the federation of national psycho-oncology societies	Editorial	In many countries around the world, especially developing countries, psychosocial oncology is not established and/or fully integrated into the care of patients with cancer. More work is needed to integrate psychosocial oncology services into comprehensive cancer centers in developing countries.
Asuzu, 2015	19	Patients and clinicians at the department of radiotherapy, University college hospital, Ibadan, Nigeria	Narrative review	At the University College Hospital in Ibadan, Nigeria, psychosocial oncology services have included screening and managing distress via one-on-one and group counseling, psychoeducation, and psychological interventions. However, more work is needed to establish a robust psychosocial oncology clinician workforce and integrate psychosocial oncology services in overall cancer care in Nigeria.
Agha, 2021	20	Multidisciplinary clinicians from social work, palliative care, and medical oncology	Qualitative	Social workers are critical to every aspect of the cancer care cycle in Nigeria. However, social workers in Nigeria may lack the necessary and up-to-date expertise to meet all the psychosocial oncology needs of patients with cancer.
Travado, 2017	21	2016 President’s plenary speakers at the annual international psychosocial oncology society meeting	Commentary	There are significant disparities in psychosocial oncology services available to patients with cancer around the world. The development of resource-stratified clinical practice guidelines for effectively managing the psychosocial needs of patients with cancer may help with the psychosocial oncological care of patients in low- and middle-income countries.

**TABLE 2:** Examples of typical patient cases referred to mental health clinicians at the national radiotherapy, oncology, and nuclear medicine center in Ghana

	Case 1	Case 2	Case 3	Case 4
Psychosocial oncology consult question	Evaluation for depressed mood	Evaluation for anxiety	Patient refusing further cancer treatment	Evaluation for depressed mood
History of present illness	60 year old female with diagnosis of stage 2, grade 2 endometrial cancer who was referred with worsening symptoms of depressed mood. Patient reports feeling sad, otherwise denies other neurovegetative symptoms. Patient denies suicidal or homicidal ideation. Patient denies psychosis or symptoms of mania.	40 year old female with a 6-month history of breast lump reporting to clinic after being referred from a secondary facility. Patient reports anxiety in the setting of lump in her breast. She is tearful during the assessment. Other psychosocial stressors entail losing a significant amount of money in an investment deal about a year ago. She denies neurovegetative symptoms. Patient denies suicidal or homicidal ideation. Patient denies psychosis or symptoms of mania.	Patient is a 52 old female with left breast cancer T2N1M0 who was referred post mastectomy.	53 year old male with renal carcinoma PT3a Grade I status post radical nephrectomy. Patient was at his baseline level of health until 4 months go when he started passing blood in his urine which eventually progressed to passing frank blood with clots. He reports sadness in the setting of physical symptoms and cancer diagnosis.
Oncologic history	Patient was well until a year prior when she noticed intermittent, purulent and foul-smelling vaginal discharge with no bleeding. After 5-months of vaginal discharge she presented to a gynecologist who discovered an abnormal fuild collection in the endometrial cavity on ultrasound. Fluid sample was sent for histopathology test which confirmed adenocarcinoma of endometrial type infiltrating 2/3 of myometrium and extending down into stroma of upper half of the cervix. Her ovary and fallopian tube showed no abnormality. She was then referred for adjuvant chemotherapy treatment.	Patient presents with right breast lump measuring 10 cm by 8 cm confirmed on mammogram with complementary ultrasonography suspicious of malignancy. Biopsy and pathology results confirm right breast invasive cancer no special type (NST) grade III, tripple negative. Clinical staging T4Bn2M0.	A lump was detected in the left breast about 2 months prior to referral during a routine breast screening exercise. Mammogram confirmed a breast mass. Pathology confirmed an infiltrating ductal carcinoma grade II. Patient was clinically staged cT2N1M0. She underwent mastectomy with oncology follow-up.	Urethrocystoscopy revealed bladder tumors with necrotic tissue Biopsy was not completed. CT scan of the abdomen showed renal cell tumor. Radical neprectomy and pathology of tumor confirmed grade I PT3 cancer of kidney.
Oncologic treatment	Adjuvant chemotherapy	Adjuvant chemotherapy	Mastectomy Brachytherapy and radiotherapy	Radical nephrectomy
Medical history	Appendicitis status post appendectomy	None	None	Radical nephrectomy
Past psychiatric history	Prior psychiatric diagnoses: None Psychiatric Hospitalizations: None Suicidal attempt: None Trauma history: None Substance use: None Outpatient psychiatric providers: None	Prior psychiatric diagnoses: None psychiatric Hospitalizations: None Suicidal attempt: None Trauma history: None Substance use: None Outpatient psychiatric providers: None	Prior psychiatric diagnoses: None Psychiatric hospitalizations: None Suicidal attempt: None Trauma history: None Substance use: None Outpatient psychiatric providers: None	Prior psychiatric diagnoses: None Psychiatric hospitalizations: None Suicidal attempt: None Trauma history: None Substance use: None Outpatient psychiatric providers: None
Social history	Born and raised: Accra Marital status: Married Family: Has two sons aged 34 and 28. Has daughter aged 30 Education: None Occupation: Trader Religious affiliation: Christian	Marital status: Separated and in the midst of divorce proceedings. Family: 10 year old daughter. Occupation: Police officer Education: Secondary school graduate Religious affiliation: Christian	Born and rasied: Accra. Marital status: Married family: Three children Education: Secondary school graduate Occupation: Not obtained Religious affiliation: Not obtained	Born and raised: Accra. Marital status: Married family: Four children. Education: College graduate Occupation: Banker Religious affiliation: Not obtained
Laboratory testing	Not obtained	Not obtained	Not obtained	Not obtained
Pertinent medications	None	None	Morphine	Tamsulosin
Physical exam	Normal physical exam	Normal physical exam	Normal physical exam	Normal physical exam
Mental status exam	Appearance: Neatly dressed Behaviour: Well-related Psychomotor activity: Calm. Speech: Normal rate, tone, prosody Mood: ‘sad’ Affect: Depressed Thought process: Linear, coherent Associations: No loosening of associations Suicidal/Homicidal Ideation: No suicidal ideation and no homicidal ideation Perceptions/Experiences: No hallucinations Insight: Fair Judgment: Fair Orientation: Oriented to person, place and time.	Appearance: Neatly dressed Behaviour: Well-related psychomotor activity: Pacing, figeting Speech: Normal rate, tone, prosody Mood: Tearful Affect: Anxious appearing Thought process: Linear, coherent Associations: No loosening of associations Suicidal/Homicidal Ideation: No suicidal ideation and no homicidal ideation Perceptions/Experiences: No hallucinations Insight: Fair Judgment: Fair Orientation: Oriented to person, place and time.	Appearance: Neatly dressed Behaviour: Withdrawn psychomotor activity: Calm Speech: Normal rate, tone, prosody Mood: Sad Affect: Depressed appearing Thought process: Linear, coherent Associations: No loosening of associations Suicidal/Homicidal Ideation: No suicidal ideation and no homicidal ideation Perceptions/Experiences: No hallucinations Insight: Fair Judgment: Fair Orientation: Oriented to person, place and time.	Appearance: Neatly dressed Behaviour: Sad psychomotor activity: Fidgeting Speech: Slowed, slurred Mood: Sad Affect: Depressed appearing Thought process: Linear, coherent Associations: No loosening of associations Suicidal/Homicidal Ideation: No suicidal ideation and no homicidal ideation Perceptions/Experiences: No hallucinations Insight: Fair Judgment: Fair Orientation: Oriented to person, place and time.
Psychological assessment tool used	Brief symptom Inventory and distress thermometer	Brief symptom Inventory and distress thermometer	Brief symptom Inventory and distress thermometer	Mini mental status exam, brief symptom Inventory and distress thermometer.
Psychiatric diagnosis	None	None	None	None
Treatment plan	Cognitive behaviour therapy (CBT) with focus on cognitive restructuring and supportive therapy	Relaxation training, CBT with focus on cognitive restructuring and supportive therapy.	Relaxation training, CBT with focus on cognitive restructuring and supportive therapy.	CBT with focus on cognitive restructuring and supportive therapy. Thought diary.

## Data Availability

Data sharing not applicable to this article as no datasets were generated or analysed during the current study.
